# Feasibility and preliminary efficacy of app-based audio tools to improve sleep health in working adults experiencing poor sleep: a multi-arm randomized pilot trial

**DOI:** 10.1093/sleep/zsad053

**Published:** 2023-03-15

**Authors:** Marcos Economides, Rhian Male, Heather Bolton, Kate Cavanagh

**Affiliations:** Science Department, Unmind Ltd, London, UK; Science Department, Unmind Ltd, London, UK; Science Department, Unmind Ltd, London, UK; School of Psychology,University of Sussex, East Sussex, UK

**Keywords:** sleep disturbance, smartphone app, digital intervention, work productivity, mental health, randomized controlled trial, feasibility, efficacy

## Abstract

**Study Objectives:**

Many adults without a diagnosed sleep disorder report poor sleep. Recently, there has been a dramatic increase in the use of app-based audio tools to aid sleep by the general public, yet there is a paucity of evidence on whether such tools are efficacious. This study evaluated the feasibility and preliminary efficacy of two categories of audio tools, comprising music and narrated stories, featured on the Unmind app.

**Methods:**

We conducted an online, parallel, multi-arm, external pilot randomized controlled trial, with two intervention arms and a waitlist (WL) control group. Participants were working adults who were screened for poor sleep. Feasibility was assessed via objective and self-report measures. Preliminary efficacy was evaluated via self-report measures of sleep disturbance, work productivity, and other mental health outcomes, captured at baseline (*t0*) and following a 4-week intervention period (*t1*), and analyzed using mixed effects models with intention-to-treat principles.

**Results:**

Three hundred participants were randomized, and 92% were retained at *t1*. 90.5% of participants completed at least one intervention session. 82.1% reported being “satisfied” or “very satisfied” with their intervention, and 84.3% rated their intervention as “good” or “excellent.” The between-group Hedges’ *g* effect size for sleep disturbance was 0.92 [0.63–1.22] and 1.09 [0.80–1.39] for the two interventions compared to the WL group.

**Conclusions:**

Both interventions are feasible and acceptable. Preliminary efficacy findings suggest that audio tools designed to aid sleep could have widespread financial and public health implications, and should be investigated in a definitive trial.

**Clinical Trial:**

International Standard Randomized Controlled Trial Number (ISRCTN), 12614821, http://www.isrctn.com/ISRCTN12614821.

## Graphical abstract

**Graphical Abstract:**
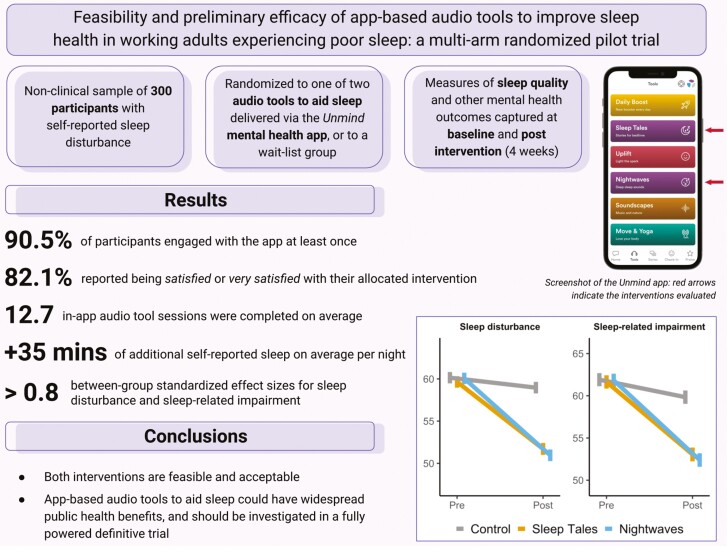

**Statement of Significance**

Poor sleep is pervasive, becoming increasingly prevalent, and has profound personal, social, economic, and public health consequences. Recently, there has been a dramatic increase in the use of app-based audio tools by the public before and during sleeping hours. These interventions are cheap, widely available, easy to use, and unlikely to cause harm, yet there is a lack of evidence regarding their efficacy and underlying mechanisms of action. This study takes the first steps to address this gap in knowledge, and pave the way for further research. We show that two categories of audio tools featured on the Unmind mental health app are both feasible and acceptable, and demonstrate preliminary efficacy across many sleep and mental health outcomes.

## Introduction

### Background and rationale

Sleep plays a vital role in maintaining physical and mental health [1–3]. Recent consensus states that adults require 7–9 hours of sleep for optimal functioning [4], yet increased demands from work, higher perceived stress, and greater use of digital technology have led to an upwards trend in sleep disturbance [5]. In the United States, 35% of adults report insufficient sleep [6], and 20% of young adults in Europe sleep an average of 90 minutes less than needed per night [7]. Moreover, worldwide, approximately one in three individuals will experience at least one symptom of insomnia, with women and older adults at higher risk [8, 9].

Insufficient sleep has been shown to adversely impact a multitude of outcomes, including cognitive performance, emotional regulation, and quality of life [2, 10]. Individuals with poor sleep are also at an elevated risk of morbidity and mortality from causes such as cancer, stroke, diabetes, and cardiovascular disease [11]. This has major implications for the workplace, with sleep-related productivity loss costing the UK economy £40.2bn a year, a figure that is set to rise to £47bn by 2030 [11], and further costs resulting from absenteeism, staff turnover, and increased healthcare expenditure [12, 13]. Moreover, whilst sleep disruption has long since been identified as a symptom of mental ill health [14, 15], recent frameworks suggest it is also a contributory causal factor in the occurrence of several mental health disorders [16, 17]. Together, this suggests an urgent need for evidence-based interventions that can improve sleep health at a population level.

There has been increased interest in the use of smartphone-based mental health apps (“MHapps”) to deliver psychosocial interventions. Relative to traditional care, MHapps are more accessible, can provide momentary support, are associated with lower costs, and can facilitate increased engagement through tailored interventions [18]. Moreover, usage of smartphones before and during sleeping hours has substantially increased, suggesting that MHapps are a prime delivery medium for sleep interventions [19]. Preliminary findings from a recent systematic review suggest that sleep interventions delivered via MHapps can attenuate sleep disorders and enhance sleep quality, and may have benefits over traditional treatment approaches [20].

To date, most evaluations of sleep interventions delivered via MHapps have focused on formal treatment programs designed for clinical populations and specific disorders, such as cognitive behavioral therapy (CBT) for insomnia [21]. Yet, many adults without a diagnosed disorder report poor sleep, and there has been a dramatic increase in the demand, availability, and media coverage of standalone, audio-based tools designed for ad hoc use by the public before and during sleeping hours [22]. Such tools often comprise relaxing soundscapes or narrated stories, with the latter being the most popular category of content used by subscribers of the well-being app *Calm* [22]. Relative to app-based CBT and other structured interventions, standalone tools may have fewer barriers to use, as they do not require active learning or significant time investment.

Although such standalone tools are widely used by individuals with disturbed sleep [22], there is a lack of empirical evidence regarding their efficacy. Some preliminary evidence suggests that music-based interventions can improve sleep quality in insomnia patients [23], though findings in nonclinical samples [24, 25], and from interventions that utilize white noise [26], have been mixed. One recent study evaluating music and narration-based sleep tools from the *Headspace* app reported partial evidence of improved sleep quality, but utilized a small study sample and a non-randomized design [27]. In addition, one randomized controlled trial (RCT) reported reductions in daytime fatigue and presleep arousal following the use of sleep-related content on the *Calm* app, but did not specifically measure sleep quality, and did not separate relaxation-based tools from mindfulness-based content [28]. Together this suggests an urgent need for further, high-quality randomized trials.

Finally, there is ambiguity regarding the potential mechanisms of action of standalone audio tools in improving sleep health. One hypothesis is that they may counteract presleep sympathetic arousal [29], or they may shift attention away from inhibitory presleep cognitive processes, such as worry [30]. Other possibilities are that they might act to replace behaviors that could otherwise increase arousal, or help to reverse negative underlying beliefs about sleep by promoting short-term sleep improvements. Understanding both the potential efficacy and mechanisms of action of such tools is needed to fully realize their utility.

### Study objectives

In this study, we conducted an external pilot RCT to evaluate the feasibility, acceptability, and preliminary efficacy of two categories of standalone, audio-based tools featured on the *Unmind* mental health app, named *Nightwaves* (*NW*; ambient music and nature sounds) and *Sleep Tales* (*ST*; narrated stories). The study recruited community-dwelling, UK-based, working adults, who were screened for poor sleep, and randomly allocated to one of two intervention arms or to a waitlist (WL) control group, for a 4-week intervention period. Consistent with guidelines on pilot trials [31, 32], the primary aim was to evaluate the feasibility of the study methods and interventions in preparation for a definitive RCT. A secondary aim was to establish the preliminary efficacy of each intervention with respect to self-report measures of sleep quality, sleep-related impairment, workplace productivity, and other mental health outcomes, including establishing between-group effect sizes and 95% CIs. At the end of the intervention period, participants in the WL group were allowed to engage freely with both interventions for a further 4 weeks, before being invited to complete a final assessment. This allowed for a preliminary assessment of intervention acceptability and efficacy under more pragmatic conditions.

## Methods

### Trial registration and ethical approval

This study received ethical approval from the University of Sussex Sciences & Technology Research Ethics Committee (ER/KC226/6), was preregistered at International Standard Randomized Controlled Trial Number 12614821, and a full study protocol was preregistered at Open Science Framework in October 2021. The authors followed the CONSORT (Consolidated Standards of Reporting) 2010 guidelines when preparing this manuscript [33], including extensions to pilot [31], and multi-arm trials [34].

### Design and setting

This study was a parallel, multi-arm, pilot RCT with pre- (*t*0) and post-intervention (*t*1) assessments. Participants were randomized to one of two interventions, accessed via the *Unmind* app, or to a WL control group, via a 1:1:1 allocation ratio, for a 4-week intervention period. Following *t1*, participants in the WL group were able to engage with both interventions freely for a further 4 weeks, after which they were invited to complete a final assessment (*t2*). Data were collected between October and December 2021, and all study procedures were conducted online.

### Participants

Participants were recruited from the Prolific online recruitment platform (https://www.prolific.co). Eligibility criteria included (1) 18 years old and above, (2) residing in the United Kingdom, (3) in full- or part-time employment, (4) probable experience of poor sleep, indicated via a score ≥7 on the Jenkins Sleep Scale [35], (5) self-reporting an interest in improved sleep, (6) fluent in English, (7) having access to the internet, (8) owning a smartphone (or tablet) and being willing to download and sign up to the *Unmind* app, (9) willingness to be randomized, and (10) having an active Prolific account. Individuals undergoing treatment for a sleep disorder with a health professional, and those reporting previous or current use of *Unmind* app, were excluded.

### Procedures and randomization

Participants were invited to complete a brief screening survey, advertised on Prolific, to determine trial eligibility. To maximize sample representativeness (and account for a proportionally higher number of young adults on the Prolific platform), separate screening adverts were created for males and females, with each being stratified further into two age groups (18–25 years, and ≥26). This ensured that ~10% of screened individuals would be in the 18–25 age group, which is broadly consistent with the general UK population (based on UK Census data, 2011).

All eligible individuals were invited to complete a baseline survey (*t*0) on a first-come-first-served basis, which automatically closed when the required sample size was met. At the end of the *t0* survey, participants were randomized to one of three study groups for a 4-week intervention period. Randomization was automated via the Qualtrics “randomizer” feature (which uses block randomization), and thus the allocation sequence was not visible to either study participants or researchers. Qualtrics software uses variable block sizes to ensure balanced groups. Participants randomized to an intervention arm were given both video and written instructions explaining how to access and engage with the interventions, and were sent weekly engagement reminders manually by a member of the research team (using Prolific’s anonymous messaging system). At the end of the intervention period, access to the interventions was withdrawn, and all participants were invited to complete a post-intervention survey (*t1*). Participants randomized to the WL group were given the opportunity to engage with both interventions freely for a further 4 weeks, before being invited to complete a final survey (*t2*). During this 4-week period, participants were sent weekly messages reminding them they had access to the interventions, though it was made clear that engagement was optional.

All surveys were hosted on Qualtrics (https://www.qualtrics.com) and included detailed online information sheets that participants were encouraged to read. Participants had to provide informed consent before being able to progress to the screening and *t0* surveys. During the *t0* survey, participants were presented with an additional video that explained the study procedures, followed by a short quiz to ensure participants understood key study details. Participants scoring <75% on the quiz were asked to rewatch the video and retake the quiz. All participants were shown the correct answers following either the first or second attempt, as applicable. Participants were paid £1 for completing the screening survey, and £10 for each trial survey (payment was not contingent upon intervention adherence).

### Interventions

*Unmind* is a digital mental health platform designed to help working adults measure, manage, and improve their mental health, and has been described in full previously [36]. One type of intervention featured on the platform comprises standalone audio exercises designed to be used ad hoc (known as *Tools*) that address different mental health or well-being topics. This study evaluated two categories of *Tools* intended to facilitate sleep, and designed to be used prior to or immediately before bed, as an aid to falling asleep, or as an aid to getting back to sleep after waking. These two categories are: (1) *Nightwaves* (*NW*; ambient music and nature sounds, each of which is 30–60 minutes in duration), and (2) *Sleep Tales* (*ST*; narrated stories, typically 15–40 minutes in duration, that include background music and/or sound cues). The content of ST tends to focus on detailed sensory descriptions of visual scenes, such as vividly describing a painting or a natural landscape. They occasionally include short breathing prompts, or deep breathing exercises, either at the start of each session or in between the descriptive storytelling, to aid relaxation. NWs on the other hand does not include any voice narration.

**Figure 1.**
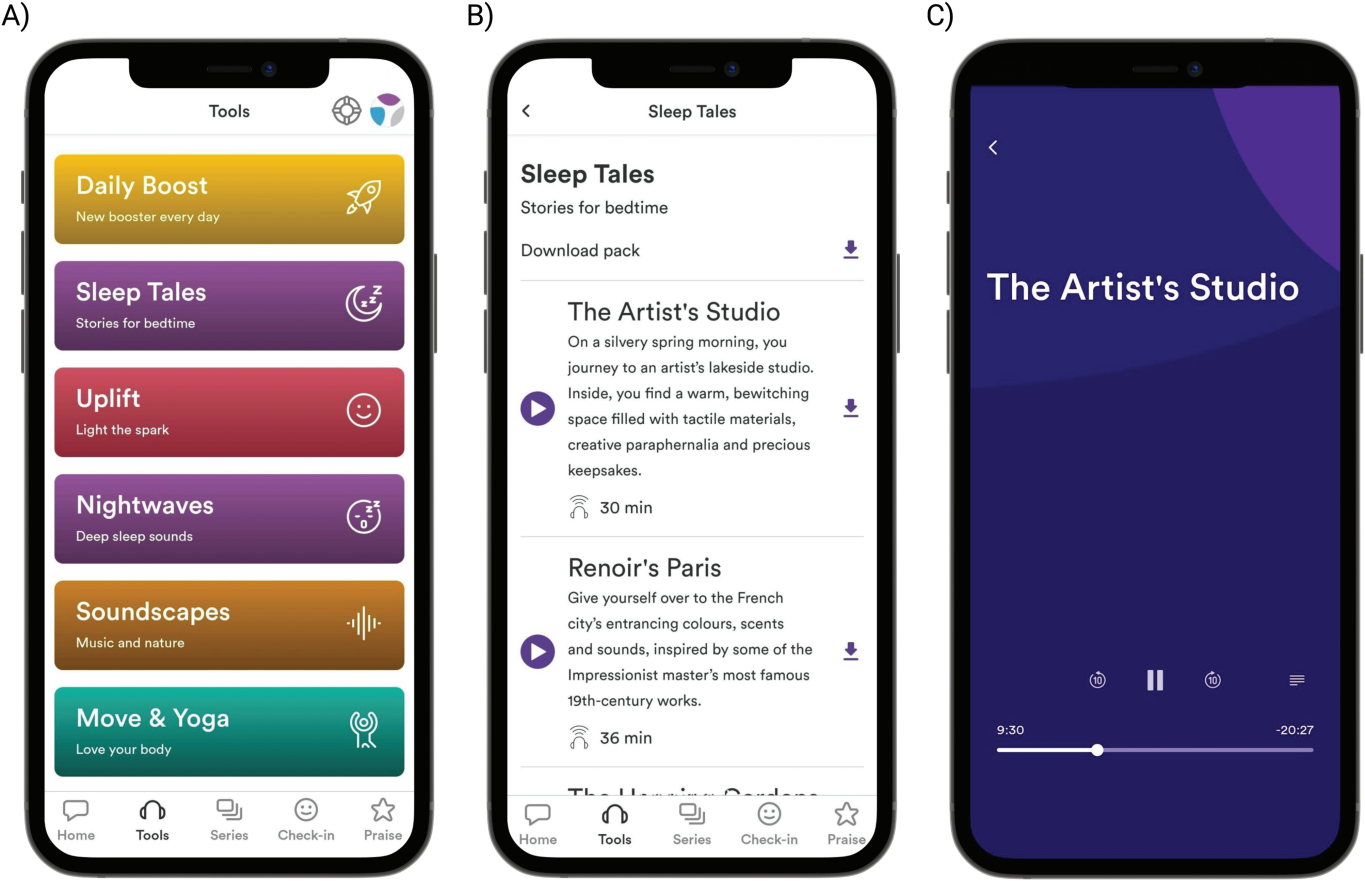
Screenshots of (A) The *Tools* homepage, (B) Example content available within the *Sleep Tales* category, and (C) An example of a *Sleep Tale* being played via the app’s built-in media player.

At the time of running this study, there were 49 *NWs* and 9 *STs* available to participants. Figure 1 includes example screenshots of both interventions as featured on the *Unmind* mobile app. *Tools* are played via a media player that allows users to play, pause, or scrub back and forth as desired. The app also includes a looping function, which when selected will continue replaying a *Tool* until stopped by the user. Participants were given access to a modified version of the app that only included their allocated intervention, and was instructed to engage with one tool of their choosing per night, with the proviso that it was acceptable to miss occasional nights. Although *Unmind* can be accessed via a web app, participants were instructed to download the mobile app (via the Apple or Google Play stores), given that the interventions are designed to be used during sleeping hours. Participants had access to versions 2.90.0–2.99.0 of the mobile app, and no major app changes or updates were launched during the study period.

### Outcomes

#### Primary outcome measures.

Primary outcomes were based on guidelines for evaluating novel health interventions, including conducting pilot trials [37, 38], and included the following:

1. Feasibility: recruitment, intervention uptake, and retention at *t1*.
2. Acceptability: intervention adherence (including the average number and length of tool sessions completed, and the proportion of participants completing an average of three tools per week, or 12 in total), participant satisfaction, and reasons for nonadherence.
3. Engagement: average number of tool sessions completed, average total duration of tool usage, self-reported conditions that prompt tool usage, self-reported pattern of tool usage, and one question adapted from section B of the Mobile App Rating Scale (MARS [39]).
4. Transferability: One item adapted from section E of the MARS.
5. Relevance: One item assessing subjective self-reported of the interventions.
6. Lasting negative effects: One item adapted from recent guidelines on assessing negative effects [37, 40]; the proportion of participants whose self-reported mental health and sleep disturbance scores (as measured by the Patient Health Questionnaire-4 [PHQ-4] and the PROMIS Sleep Disturbance Short Form [SD-SF]) reliably deteriorated from *t0* to *t1* (as recommended by [40]).

#### Secondary outcome measures.

Preregistered secondary outcomes included the following self-report measures, completed at *t0* and *t1* for all study arms, and *t2* for the WL group only.

#### PROMIS sleep disturbance short form 8a.

The SD-SF measures perceived difficulties with getting to or staying asleep, as well as perceived adequacy of and satisfaction with sleep, and is universal rather than disorder-specific [41]. Respondents are asked to rate each of the eight items on a 5-point Likert scale (from 0 to 5) over a 7-day recall period. Total scores range from 8 to 40 with higher scores indicating greater sleep disturbance. Raw scores are converted to standardized T-scores with a mean of 50 and standard deviation (SD) of 10. The SD-SF has excellent reliability (0.90 or above), and greater measurement precision than the widely used Pittsburgh Sleep Quality Index (PSQI) [41].

#### PROMIS sleep-related impairment short form 8a.

The PROMIS sleep-related impairment short form 8a (SRI-SF) captures self-reported perceptions of alertness, sleepiness, and tiredness during usual waking hours, as well as sleep-related functional impairments during wakefulness, and is universal rather than disorder-specific [41]. The response options, recall period, scoring, and interpretation are equivalent to the SD-SF. The SRI-SF has excellent reliability (0.90 or above), and greater measurement precision than both the Epworth Sleepiness Scale and PSQI [41].

#### Sleep latency and duration.

Participants were asked to complete the first two questions of the Medical Outcomes Study Sleep Scale [42] which ask about (1) the time taken to fall asleep, and (2) the number of hours of sleep each night, using a 4-week recall period.

#### Work productivity and activity impairment.

The work productivity and activity impairment (WPAI) is a 6-item self-report measure of health-related work productivity loss for the employed population [43]. This study used the specific health problem version (WPAI:SHP), with “poor sleep” being the target problem. The WPAI uses a 1-week recall period. The WPAI produces four scores (expressed as percentage impairment) that capture (1) absenteeism (work time missed), (2) presenteeism (impairment at work/ reduced on-the-job effectiveness), (3) work productivity loss (overall work impairment/ absenteeism plus presenteeism), and (4) activity impairment. The WPAI has sufficient construct validity and test–retest reliability (with correlation coefficients ranging from 0.71 to 0.87 for overall productivity loss).

#### Short Warwick-Edinburgh mental well-being scale.

The Short Warwick-Edinburgh mental well-being scale (SWEMWBS) is designed to measure aspects of mental well-being that relate predominantly to functioning, and uses a 2-week recall period [44]. Respondents are asked to rate each item on a 5-point Likert scale from 1 = “None of the time” to 5 = “All of the time.” Raw scores are transformed into metric scores ranging from 7 to 35, with higher scores indicating greater positive mental well-being. The SWEMWBS has high internal consistency (*a* = 0.84 [45]).

#### Unmind Index.

The Unmind Index is a 26-item measure designed to capture key components of positive mental well-being and specific symptoms of mental ill health, using a 2-week recall period [46]. Respondents are asked to rate each item on a 6-point Likert scale from 0 = “No days” to 5 = “Every day.” The Unmind Index comprises a general factor (the *Overall Score*) and seven subscales, each of which is scored by normalizing and standardizing raw mean scores to population norms, with a resulting mean of 100 and SD of 15. The *Overall Score* is calculated as the mean of the subscale scores, and also has a mean of 100, with higher scores indicating less symptomatology and greater well-being. The Unmind Index shows excellent internal consistency for the *Overall Score* (McDonald’s hierarchical omega = 0.92).

#### Patient Health Questionnaire-4.

The PHQ-4 is a brief, 4-item measure designed to capture core symptoms of depression and anxiety over a 2-week recall period [47]. Respondents are asked to rate each item on a 4-point Likert scale ranging from 0 = “Not at all,” to 3 = “Nearly every day.” Scores are interpreted as follows: normal (0–2), mild (3–5), moderate (6–8), and severe (9–12). The PHQ-4 has strong psychometric properties including good internal consistency (*a* = 0.85).

### Other outcomes

#### Jenkins Sleep Quality Scale (JSS).

The JSS is a brief, 4-item measure that evaluates the frequency and intensity of certain sleep difficulties [35], and was used in the current study to screen for the presence of disturbed sleep during recruitment. Participants are asked to indicate the frequency with which they have experienced specific sleep problems over the past month, using a 5-point Likert scale (0 = “Not at all,” 1 = “1–3 days,” 2 = “4–7 days,” 3 = “8–14 days,” 4 = ‘15–21 days, 5 = “22–31 days”). Total scores range from 0 to 20, with higher scores indicating greater sleep disturbance. A cutoff of ≥7 was used to identify individuals with self-reported disturbed sleep, based on previous receiver operator analyses [48, 49].

#### Demographics questionnaire.

Participants were presented with a demographics questionnaire at *t0* designed to capture age, sex, gender identity, ethnicity, education, employment status, workplace industry, and other characteristics related to sleep (see Table 1).

**Table 1.** Participant demographics and baseline characteristics

Variable	Overall (*n* = 300)	Study arm		
		WL (*n* = 101)	ST (*n* = 99)	NW (*n* = 100)
Age (years)				
Mean (SD)	35.5 (9.0)	37.0 (9.1)	34.9 (8.9)	34.4 (8.6)
Range	18.0, 64.0	19.0, 61.0	21.0, 64.0	18.0, 59.0
Sex, *n* (%)				
Female	215 (71.7)	70 (69.3)	70 (70.7)	75 (75.0)
Male	85 (28.3)	31 (30.7)	29 (29.3)	25 (25.0)
Ethnicity, *n* (%)				
White	274 (91.3)	91 (90.1)	88 (88.9)	95 (95.0)
Black	2 (0.7)	1 (1.0)	1 (1.0)	0 (0.0)
Mixed	9 (3.0)	2 (2.0)	4 (4.0)	3 (3.0)
Asian	13 (4.3)	6 (5.9)	6 (6.1)	1 (1.0)
Other	2 (0.7)	1 (1.0)	0 (0.0)	1 (1.0)
Education, *n* (%)				
None	3 (1.0)	1 (1.0)	1 (1.0)	1 (1.0)
High School	118 (39.3)	41 (40.6)	36 (36.4)	41 (41.0)
University degree	132 (44.0)	45 (44.6)	45 (45.5)	42 (42.0)
Postgraduate degree	47 (15.7)	14 (13.9)	17 (17.2)	16 (16.0)
Employment status, *n* (%)				
Full-time	200 (66.7)	75 (74.3)	65 (65.7)	60 (60.0)
Part-time	75 (25.0)	23 (22.8)	20 (20.2)	32 (32.0)
Self-employed/Contractor	25 (8.3)	3 (3.0)	14 (14.1)	8 (8.0)
Employment industry, *n* (%)				
Agriculture/Forestry/Mining	3 (1.0)	0 (0.0)	0 (0.0)	3 (3.0)
Industrials	14 (4.7)	5 (5.0)	5 (5.1)	4 (4.0)
Energy/Utilities	4 (1.3)	1 (1.0)	2 (2.0)	1 (1.0)
Transport/Logistics	9 (3.0)	4 (4.0)	3 (3.0)	2 (2.0)
Media/Creative Industries	14 (4.7)	4 (4.0)	6 (6.1)	4 (4.0)
Data/Telecom	16 (5.3)	6 (5.9)	8 (8.1)	2 (2.0)
Healthcare	40 (13.3)	13 (12.9)	11 (11.1)	16 (16.0)
Education	43 (14.3)	15 (14.9)	15 (15.2)	13 (13.0)
Life Sciences	5 (1.7)	1 (1.0)	3 (3.0)	1 (1.0)
Retail	26 (8.7)	7 (6.9)	10 (10.1)	9 (9.0)
Hospitality/Leisure/Travel	20 (6.7)	8 (7.9)	5 (5.1)	7 (7.0)
Public/Social Service	30 (10.0)	13 (12.9)	5 (5.1)	12 (12.0)
Finances/Insurance/Real Estate	29 (9.7)	9 (8.9)	10 (10.1)	10 (10.0)
Professional Services	17 (5.7)	5 (5.0)	9 (9.1)	3 (3.0)
Other	30 (10.0)	10 (9.9)	7 (7.1)	13 (13.0)
Long term leave, *n* (%)				
Sick leave	8 (2.7)	3 (3.0)	3 (3.0)	2 (2.0)
Other leave	9 (3.0)	0 (0.0)	4 (4.0)	5 (5.0)
No	283 (94.3)	98 (97.0)	92 (92.9)	93 (93.0)
Attended therapy (past 6 mo), *n* (%)				
Yes	19 (6.3)	5 (5.0)	10 (10.1)	4 (4.0)
No	281 (93.7)	96 (95.0)	89 (89.9)	96 (96.0)
Mental health app use (past 6 mo), *n* (%)				
Yes	29 (9.7)	9 (8.9)	14 (14.1)	6 (6.0)
No	271 (90.3)	92 (91.1)	85 (85.9)	94 (94.0)
Children in care, *n* (%)				
Yes	116 (38.7)	39 (38.6)	37 (37.4)	40 (40.0)
No	184 (61.3)	62 (61.4)	62 (62.6)	60 (60.0)
Bed partner arrangements, n (%)				
No bed partner	98 (32.7)	35 (34.7)	31 (31.3)	32 (32.0)
Partner sleeps in different bed	21 (7.0)	9 (8.9)	6 (6.1)	6 (6.0)
Partner sleeps in same bed	181 (60.3)	57 (56.4)	62 (62.6)	62 (62.0)
Perform shift work, *n* (%)				
Yes	31 (10.3)	12 (11.9)	9 (9.1)	10 (10.0)
No	269 (89.7)	89 (88.1)	90 (90.9)	90 (90.0)
Use of sleep remedies, *n* (%)				
Yes	16 (5.3)	4 (4.0)	9 (9.1)	3 (3.0)
No	284 (94.7)	97 (96.0)	90 (90.9)	97 (97.0)
Past sleep diagnosis, *n* (%)				
Yes	2 (0.7)	0 (0.0)	1 (1.0)	1 (1.0)
No	298 (99.3)	101 (100.0)	98 (99.0)	99 (99.0)
Alcohol intake, *n* (%)				
Never	57 (19.0)	19 (18.8)	21 (21.2)	17 (17.0)
Monthly or less	100 (33.3)	31 (30.7)	32 (32.3)	37 (37.0)
Several per month	74 (24.7)	23 (22.8)	23 (23.2)	28 (28.0)
Several per week	61 (20.3)	27 (26.7)	20 (20.2)	14 (14.0)
Almost daily	8 (2.7)	1 (1.0)	3 (3.0)	4 (4.0)
Other factors impacting sleep, *n* (%)				
Yes	104 (34.7)	35 (34.7)	34 (34.3)	35 (35.0)
No	196 (65.3)	66 (65.3)	65 (65.7)	65 (65.0)

#### Feedback questionnaire.

Participants were presented with a feedback questionnaire at *t1* (or *t2* for the WL group) designed to capture intervention engagement, satisfaction, and other feasibility outcomes, which included questions adapted from the MARS.

### Progression criteria

Progression criteria for a definitive trial were predefined as per formal guidelines [50] and were as follows: (1) full study recruitment within 1 month, (2) ≥80% of participants in the intervention arms signing up to *Unmind*, (3) ≥50% of participants reporting being “satisfied” or “very satisfied” with their allocated intervention and rating the quality as “good” or “excellent,” and (4) the 95% CI on between-group effect sizes (*NW* or *ST* vs. WL) for self-reported sleep quality (measured via the SD-SF) including at least a small effect (Hedges’ *g* = 0.2). The latter two criteria were evaluated for each intervention arm separately.

### Sample size

This study was powered for CIs on the feasibility outcomes. A sample size calculation indicated that approximately 100 participants were required to estimate feasibility outcomes with a margin of error of ≤10% (based on a conservative population proportion of 50% for retention and adherence, and a 95% CI). This is consistent with previous guidelines suggesting that 60 to 100 participants per intervention arm are optimal for estimating binary outcomes in pilot RCTs [51]. We, therefore, aimed to recruit 300 participants in total. Previous data suggested that approximately 20% of individuals from the general population would meet our criterion for disturbed sleep (scoring ≥7 on the JSS [52]). On this basis, we estimated that approximately 2000 individuals would need to be screened to achieve a final sample size of 300.

### Statistical analyses

#### Primary analyses.

Descriptive statistics were used to report on demographics and primary outcomes. Categorical data were reported as proportions in each response category, and Fisher’s exact test of independence was used to compare responses and attrition between intervention arms (two-tailed). Logistic regression was used to test for changes in self-reported sleep onset latency (captured as a categorical variable) with predictors “group,” “timepoint,” and their interaction included in the model (*P* values <0.05 were considered significant). Objective in-app usage data was provided by *Unmind*. Since the intervention sessions are long and designed to be used flexibly, an individual session was characterized as complete if the total play time was ≥5 minutes. Since *Tools* can be looped or replayed, we also calculated the number of intervention sessions played based on duration (where a single session looped once would count as two plays), and the total summed duration of *Tool* usage across the intervention period. Finally, we report the proportion of participants who completed ≥12 intervention sessions in total, which was the predefined threshold for per protocol engagement.

#### Secondary analyses.

Change in secondary outcome measures from *t0* to *t1* was analyzed using linear mixed-effects models (LMEs) with restricted maximum likelihood estimation (via the “lmerTest” package in R) and intention-to-treat principles. Each model included a within-participant factor “time” (with levels: *t0* and *t1*), a between-participant factor “group” (*ST*, *NW*, or *WL*), and their interaction as fixed effects, and a separate baseline for each participant (random intercepts). For each outcome, we report the estimated marginal means (EMMs) with standard errors (SE) for each time point and group, within-group contrasts comparing the change in scores from *t0* to *t1* (with SE and *P* values), and between-group *P* values for interaction effects (*NW* or *ST* versus *WL*), with values <.05 being considered significant. We also report a standardized effect size (Hedges’ *g* with 95% CI) for each between-group contrast. Hedges’ *g* was calculated using EMMs (as opposed to raw data, which require the use of complete cases only) and pooled SDs, using equations 1 and 2 from Nakagawa and Cuthill, and equations 15 and 16 to calculate 95% CIs [53].

To analyze changes in secondary outcomes in the WL group from *t1* to *t2*, models were adjusted to only include a single within-subject factor “time” (with levels: *t1* and *t2*). We thus report on the main effect of time, with EMMs and SE for each time point. Hedges’ *g* was calculated as described above, but was based on the mean difference in scores between *t1* and *t2*, and using equation 18 from Nakagawa and Cuthill to calculate standard error [53].

Due to a technical error, we were unable to link individual questionnaire responses to objective engagement data, and were thus unable to conduct per-protocol analyses or exploratory dose–response analyses.

## Results

### Participants

Participants' demographics and other baseline variables are presented in Table 1. The sample was 71.7% female, 91.3% White, and had a mean age of 35.5 years (SD = 9.0, range = 18–64). On average, participants scored approximately one SD above population norms for sleep disturbance and sleep-related impairment at baseline, and reported sleeping a median of 6 hours 15 minutes per night (SD = 1 hour 6 minutes); 45 minutes less than the lower bound of the healthy 7–9 hours range [4]. 193 (64.3%) participants reported a sleep onset latency of ≥45 minutes. Participants also scored between 0.5–1 SD below population norms on measures of mental health and well-being, and reported high rates of presenteeism and activity-related impairment due to poor sleep. Despite this, self-reported absenteeism was low, and only two participants had a previously diagnosed sleep disorder.

### Primary outcomes

#### Enrollment and retention.

**Figure 2.**
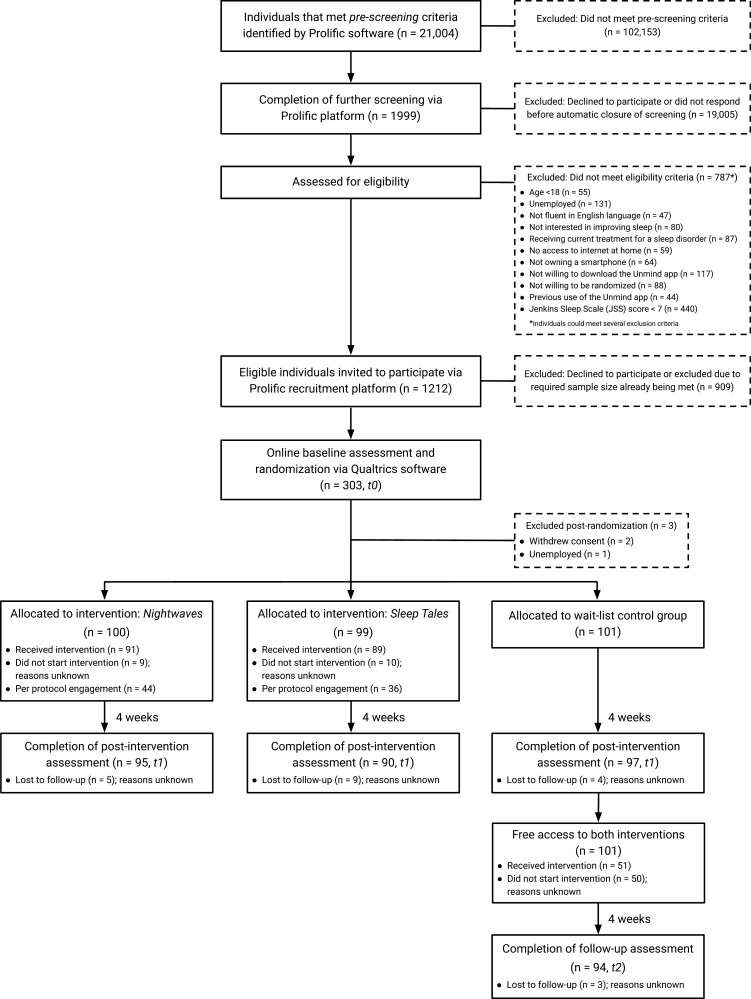
CONSORT chart of participant flow through the study.

The screening stage and main trial were both fully enrolled within 48 hours of launching the respective study adverts on Prolific. The participant flow through the trial is shown in Figure 2. Of the 1999 participants who completed screening, 1212 were eligible, of which 303 completed a baseline assessment. Two participants withdrew post-randomization, and one self-reported being unemployed, leaving 300 participants in the analysis. 282 (94%) participants were retained at *t1*, and there was no significant difference in attrition between groups (*p* = 0.318). 91 (90.1%) participants from the WL group were retained at *t2*.

#### Engagement and satisfaction.

A summary of objective intervention engagement is shown in Table 2. Of the 199 participants randomized to an intervention arm, 180 (90.5%) signed up for Unmind and completed at least one session. On average, participants completed a session in the app on 12.7 (SD = 10) separate occasions. When calculating the number of intervention sessions played based on duration, this increased to 25.3 (SD = 23.5) sessions, suggesting that participants may have frequently replayed the same session. Total session playtime across the 4-week intervention period averaged 10.9 hours (SD = 10.3). Of the 180 participants who used the app, 80 (44.4%) engaged on ≥12 separate occasions (the predefined threshold for per protocol engagement). Participants mostly commonly reported using the intervention sessions to help fall asleep (53.7% of all multi-select responses), followed by relaxing shortly before going to bed (30.5%), and less frequently as an aid to get back to sleep after waking in the night (14.7%).

**Table 2.** Summary of objective intervention engagement for the two study interventions and overall

Variable	Study arm			
	Overall (*n* = 180)	ST (*n* = 89)	NW (*n* = 91)	WL (*n* = 51)
Individual sessions completed				
Mean (SD)	12.7 (10.0)	12.0 (8.5)	13.5 (11.3)	6.5 (6.5)
Range	1.0, 57.0	1.0, 29.0	1.0, 57.0	1,0, 30.0
Sessions based on length				
Mean (SD)	25.3 (23.5)	26.0 (26.3)	24.6 (20.5)	13.7 (17.2)
Range	0.2, 146.8	0.4, 146.8	0.2, 109.7	0.3, 81.5
Total playtime (in hours)				
Mean (SD)	10.9 (10.3)	8.9 (8.9)	12.9 (11.1)	6.2 (8.0)
Range	0.1, 56.4	0.1, 56.4	0.1, 54.9	0.1, 42.6
Per protocol engagement (*n*, %)	80 (44%)	36 (40%)	44 (48%)	10 (20%)

Self-reported barriers to intervention engagement, and factors that made participants more or less likely to engage with the interventions on specific nights, are reported in Supplementary Table S1. Barriers to overall engagement included sleep naturally improving, a change in routine or circumstance, not perceiving the interventions to be helpful, a lack of time, or other reasons (with forgetting to engage being frequently cited). Participants were more likely to engage with the interventions on nights when they had a busy mind or racing thoughts, were not feeling tired or ready for sleep, were feeling more stressed or anxious than normal, or had slept badly on previous nights. Barriers to engaging on specific nights included forgetting, feeling ready to sleep without any aids, not having time, having a change in sleeping arrangements, and practical reasons (such as a low mobile battery).

Post-intervention feedback ratings are shown in Table 3. 82.1% of participants reported they were either “satisfied” (45.1%) or “very satisfied” (37.0%) with their allocated intervention, and 84.3% rated the quality of their intervention as either “good” (43.5%) or “excellent” (40.8%). When asked if they would recommend the interventions to people who might benefit from them, 51.7% of participants said they would recommend them to “many people” or to “everyone.” Ratings did not significantly differ between intervention arms.

**Table 3.** Post-intervention feedback ratings at *t*1. *P* values are from Fisher’s exact tests comparing ratings between intervention arms

Feedback question (*n*, %)		Study arm		*P* value
	Overall (*n* = 185)	ST (*n* = 90)	NW (*n* = 95)	
Was the Unmind app and intervention easy to use?				0.6
No	0 (0.0)	0 (0.0)	0 (0.0)	
Useable after a lot of time and effort	0 (0.0)	0 (0.0)	0 (0.0)	
Useable after some time and effort	9 (4.9)	5 (5.6)	4 (4.3)	
Easy to learn	56 (30.4)	30 (33.3)	26 (27.7)	
Able to use app immediately	119 (64.7)	55 (61.1)	64 (68.1)	
Would you recommend the intervention to people who might benefit from it?				0.8
Not at all	0 (0.0)	0 (0.0)	0 (0.0)	
Very few people	22 (12.0)	10 (11.1)	12 (12.8)	
Several people	67 (36.4)	36 (40.0)	31 (33.0)	
Many people	50 (27.2)	24 (26.7)	26 (27.7)	
Everyone	45 (24.5)	20 (22.2)	25 (26.6)	
Do you agree that the intervention felt relevant to your personal situation and needs?				0.3
Strongly agree	39 (21.2)	17 (18.9)	22 (23.4)	
Agree	102 (55.4)	47 (52.2)	55 (58.5)	
Neither	29 (15.8)	19 (21.1)	10 (10.6)	
Disagree	11 (6.0)	6 (6.7)	5 (5.3)	
Strongly disagree	3 (1.6)	1 (1.1)	2 (2.1)	
Would you agree with the statement “I experienced lasting bad effects from use of the intervention?”				0.8
Strongly agree	1 (0.5)	0 (0.0)	1 (1.1)	
Slightly agree	1 (0.5)	1 (1.1)	0 (0.0)	
Not sure	9 (4.9)	4 (4.4)	5 (5.3)	
Slightly disagree	10 (5.4)	6 (6.7)	4 (4.3)	
Strongly disagree	163 (88.6)	79 (87.8)	84 (89.4)	
How satisfied are you with the intervention?				0.3
Very satisfied	68 (37.0)	28 (31.1)	40 (42.6)	
Satisfied	83 (45.1)	43 (47.8)	40 (42.6)	
Neither	24 (13.0)	15 (16.7)	9 (9.6)	
Dissatisfied	9 (4.9)	4 (4.4)	5 (5.3)	
How would you rate the quality of the intervention?				0.078
Poor	1 (0.5)	1 (1.1)	0 (0.0)	
Okay	28 (15.2)	15 (16.7)	13 (13.8)	
Good	80 (43.5)	45 (50.0)	35 (37.2)	
Excellent	75 (40.8)	29 (32.2)	46 (48.9)	

#### Negative effects.

One participant randomized to ST “slightly agreed” that the intervention resulted in lasting negative effects. This individual reported waking up in the middle of night and having trouble getting back to sleep. Qualitative feedback suggested they would have preferred music-based rather than narration-based tools. Similarly, one participant randomized to NW “strongly agreed” that the intervention resulted in lasting negative effects. This individual reported experiencing headaches and worsened sleep following intervention use. Qualitative feedback suggested that the repetitive nature of the sounds featured in the NW tools resulted in increased anxiety. A further nine participants selected “not sure” when asked whether the interventions resulted in negative effects. However, analysis of secondary outcomes showed increases in sleep duration, and decreases in sleep disturbance, sleep-related impairment, and symptoms of depression and anxiety in this subgroup, suggesting they may have misunderstood the question.

When investigating reliable deterioration in outcome measures from *t0* to *t1* (for the SD-SF, SRI-SF, SWEMWBS, and PHQ-4), the proportion of participants reporting deterioration ranged from 6.4% to 12.6% for the control group, and 1.1% to 4% for the intervention groups (see Table 4). Together, this suggests that the interventions pose very minimal risk of unwanted effects, but may in rare cases be unsuited to specific individuals.

**Table 4.** Participants who reported reliable deterioration from *t0* to *t1*, based on reliable change index

Outcome	*n*	Study arm		
		WL	ST	NW
SD-SF, *n* (%)	273			
No		88 (93.6)	87 (97.8)	89 (98.9)
Yes		6 (6.4)	2 (2.2)	1 (1.1)
SRI-SF, *n* (%)	274			
No		84 (90.3)	86 (96.6)	90 (97.8)
Yes		9 (9.7)	3 (3.4)	2 (2.2)
SWEMWBS, *n* (%)	277			
No		88 (90.7)	89 (98.9)	88 (97.8)
Yes		9 (9.3)	1 (1.1)	2 (2.2)
PHQ-8, *n* (%)	243			
No		76 (87.4)	72 (96.0)	79 (97.5)
Yes		11 (12.6)	3 (4.0)	2 (2.5)

### Secondary outcomes

Changes in secondary outcomes were evaluated using intention-to-treat analyses. Table 5 shows EMMs for each outcome measure (by group and timepoint), pre–post within-group contrast estimates, between-group Hedge’s *g* effect sizes, and *P* values for each group x time interaction effect. For completeness, we report raw scores at *t0* and *t1* from complete cases only in Supplementary Table S2, which are largely equivalent to the EMMs. Both study interventions resulted in significant between-group improvements for sleep disturbance, sleep-related impairment, total sleep duration, symptoms of depression and anxiety, and mental well-being (see Table 5 for *P* values). Standardized, between-group Hedge’s *g* effect sizes were large for both sleep disturbance (ST: *g* = 0.92, 95% CI [0.63–1.22]; NW: *g* = 1.09, 95% CI [0.80–1.39]) and sleep-related impairment (ST: *g* = 0.80, 95% CI [0.51–1.09]; NW: *g* = 0.91, 95% CI [0.62–1.20]), moderate for total sleep duration (ST: *g* = 0.56, 95% CI [0.27–0.84]; NW: *g* = 0.57, 95% CI [0.29–0.85]), and in the moderate–large range for all other mental health and well-being outcomes (see Table 5). Participants in the intervention groups reported sleeping an additional 35 minutes (SD = 6 minutes) a night at *t1* compared to *t0*.

**Table 5.** Estimates marginal means (ITT sample) from linear mixed effects models with standardized effect sizes

Outcome	Estimates (mean, SE)			*P* value (Within-group)	*P* value (Between-group)	Hedges *g* [95% CI] (Between-group)
	*t0*	*t1*	*t0 – t1*			
**SD-SF**						
Control	60.1 (0.68)	59.0 (0.69)	1.18 (0.72)	0.103		
Sleep Tales	59.6 (0.68)	51.7 (0.71)	7.94 (0.74)	<0.001	<0.001	0.92 [0.63–1.22]
Nightwaves	60.1 (0.68)	51.0 (0.70)	9.11 (0.73)	<0.001	<0.001	1.09 [0.80–1.39]
**SRI-SF**						
Control	61.9 (0.78)	59.8 (0.80)	2.08 (0.80)	0.011		
Sleep Tales	61.6 (0.79)	53.1 (0.82)	8.59 (0.83)	<0.001	<0.001	0.80 [0.51–1.09]
Nightwaves	61.8 (0.79)	52.4 (0.80)	9.44 (0.81)	<0.001	<0.001	0.91 [0.62–1.20]
**Sleep duration**						
Control	6.20 (0.11)	6.26 (0.11)	0.06 (0.09)	0.506		
Sleep Tales	6.37 (0.11)	6.96 (0.11)	0.59 (0.10)	<0.001	<0.001	0.56 [0.27–0.84]
Nightwaves	6.38 (0.11)	6.97 (0.11)	0.59 (0.09)	<0.001	<0.001	0.57 [0.29–0.85]
**SWEMWBS**						
Control	19.6 (0.31)	19.6 (0.31)	0.00 (0.30)	0.996		
Sleep Tales	19.3 (0.31)	21.3 (0.32)	2.00 (0.31)	<0.001	<0.001	0.66 [0.37–0.94]
Nightwaves	20.1 (0.31)	22.1 (0.32)	2.01 (0.30)	<0.001	<0.001	0.67 [0.38–0.95]
**PHQ-4**						
Control	4.63 (0.30)	4.87 (0.31)	-0.23 (0.27)	0.392		
Sleep Tales	5.09 (0.31)	3.70 (0.31)	1.39 (0.28)	<0.001	<0.001	0.59 [0.30–0.87]
Nightwaves	4.82 (0.30)	3.00 (0.31)	1.82 (0.27)	<0.001	<0.001	0.75 [0.46–1.04]
**Unmind Index**						
Control	92.9 (0.94)	93.1 (0.95)	0.23 (0.86)	0.784		
Sleep Tales	92.9 (0.95)	101.0 (0.98)	8.13 (0.88)	<0.001	<0.001	0.91 [0.62–1.20]
Nightwaves	94.3 (0.94)	102.5 (0.96)	8.17 (0.86)	<0.001	<0.001	0.92 [0.63–1.21]
**WPAI**						
* Absenteeism*						
Control	3.82 (0.85)	3.91 (0.87)	-0.09 (1.09)	0.932		
Sleep Tales	3.31 (0.87)	1.49 (0.90)	1.82 (1.12)	0.105	0.221	0.17 [−0.12–0.45]
Nightwaves	4.26 (0.86)	1.37 (0.88)	2.89 (1.10)	0.009	0.055	0.27 [−0.01–0.55]
* Presenteeism*						
Control	45.0 (2.20)	40.8 (2.24)	4.29 (2.36)	0.069		
Sleep Tales	44.4 (2.22)	24.6 (2.31)	19.9 (2.43)	<0.001	<0.001	0.65 [0.37–0.94]
Nightwaves	41.7 (2.21)	29.6 (2.26)	12.1 (2.38)	<0.001	0.021	0.33 [0.05–0.61]
* Total work impairment*						
Control	46.6 (2.27)	42.3 (2.31)	4.33 (2.33)	0.065		
Sleep Tales	46.0 (2.30)	25.5 (2.38)	20.8 (2.41)	<0.001	<0.001	0.70 [0.41–0.98]
Nightwaves	44.7 (2.29)	30.3 (2.33)	14.4 (2.36)	<0.001	0.003	0.43 [0.15–0.71]
* Activity impairment*						
Control	51.4 (2.21)	47.0 (2.24)	4.41 (2.28)	0.055		
Sleep Tales	52.7 (2.23)	33.6 (2.31)	19.1 (2.35)	<0.001	<0.001	0.63 [0.35–0.92]
Nightwaves	54.1 (2.22)	32.7 (2.26)	21.4 (2.30)	<0.001	<0.001	0.74 [0.45–1.03]

Both interventions also significantly reduced sleep onset latency relative to the WL group. At *t0*, a total of 65.7% of participants in the ST arm and 73.0% in the NW arm reported a sleep onset latency of >30 minutes, which dropped to 33.3% and 34.7% at *t1*, respectively (see Table 6). Logistic regression with group x time interactions revealed that these changes were statistically significant for both *ST* (*b* = −1.29, SE = 0.59, *p* = 0.029) and *NW* (*b* = −1.18, SE = 0.59, *p* = 0.048), relative to the WL group. Both interventions also resulted in significant between-group improvements in sleep-related presenteeism, total work impairment, and daily activity impairment, but not absenteeism (ST: *P* = 0.221; NW: *p* = 0.055; see Table 5). However, absenteeism rates were low at baseline. Between-group Hedge’s *g* effect sizes were in the small–moderate range for presenteeism, total work impairment, and daily activity impairment (see Table 5).

**Table 6.** Self-reported sleep onset latency for each study arm and timepoint.

Group	Timepoint	Sleep onset latency (*n*, %)				
		0–15 min	16–30 min	31–45 min	46–60 min	>60 min
WL	*t0* (*n* = 101)	13 (12.9)	33 (32.7)	22 (21.8)	14 (13.9)	19 (18.8)
	*t1* (*n* = 97)	16 (16.5)	37 (38.1)	20 (20.6)	13 (13.4)	11 (11.3)
	*t2* (*n* = 94)	28 (29.8)	34 (36.2)	18 (19.1)	9 (9.6)	5 (5.3)
ST	*t0* (*n* = 99)	8 (8.1)	26 (26.3)	26 (26.3)	15 (15.2)	24 (24.2)
	*t1* (*n* = 90)	27 (30.0)	33 (36.7)	19 (21.1)	4 (4.4)	7 (7.8)
NW	*t0* (*n* = 100)	8 (8.0)	19 (19.0)	37 (37.0)	15 (15.0)	21 (21.0)
	*t1* (*n* = 95)	26 (27.4)	36 (37.9)	19 (20.0)	8 (8.4)	6 (6.3)

#### Progression criteria.

All predefined trial progression criteria were met, suggesting that a definitive trial is warranted.

#### WL group follow-up.

Of the 101 participants randomized to the WL group at baseline, 94 (93.1%) were retained at *t2.* Following the WL period, 51 participants (50.5%) signed up for the Unmind app and completed at least one intervention session. On average, participants completed a session in the app on 6.5 (SD = 6.5) separate occasions (see Table 3). When calculating the number of intervention sessions played based on duration, this increased to 13.7 (SD = 17.2). Total session playtime across the 4-week follow-up period averaged 6.2 hours (SD = 8.0).

Feedback ratings and preliminary efficacy findings from the WL group are presented in (Supplementary Materials Tables S3–S4), and engagement data is included in Table 3. Briefly, 14 participants (27.5%) engaged with ST only, 12 (23.5%) engaged with NW only, and 25 (49.0%) engaged with both interventions. Of those who self-reported engaging with both interventions, 60.0% expressed a preference for NW, 34.0% had a preference for ST, and 6.0% had no preference. Participants most frequently reported using the intervention sessions to help fall asleep (51.8% of all multi-select responses), followed by relaxing shortly before going to bed (30.9%), and less frequently as an aid to get back to sleep after waking in the night (14.5%). The primary barrier to engagement was forgetting to use the interventions. Of those who self-reported using Unmind, 78.4% rated the quality of the interventions as “good” (47.3%) or “excellent” (31.1%), and 75.7% were either “satisfied” (47.3%) or “very satisfied” (28.4%) with the interventions.

Preliminary efficacy findings were largely consistent with the between-group RCT findings, with LMEs revealing significant *t1* to *t2* improvements across all secondary outcome measures, except for absenteeism (*p* = 0.618). Within-group Hedges’ *g* effect sizes were in the moderate-large range for sleep disturbance and sleep-related impairment, and either small or moderate for all other secondary outcomes (see Supplementary Table S4).

## Discussion

Many individuals without a diagnosed disorder experience poor sleep, and this can have serious health and economic consequences. Recently, there has been surge in use of smartphone-based audio tools to aid sleep by the general public, yet little is known about the effectiveness of such tools. This study reports on the feasibility and preliminary efficacy of two categories of audio-based tools for sleep, featured on the *Unmind* MHapp, when delivered to working adults with sleep disturbance. The study methods and interventions were found to be feasible, and all preregistered criteria for progression to a definitive trial were met.

We were able to rapidly recruit a large sample of participants from the public who self-reported poor sleep at baseline using a validated screening measure. Participants self-reported significantly higher sleep disturbance and sleep-related impairment than population norms at baseline, including 40% impaired productivity whilst working due to poor sleep, despite not having a diagnosed sleep disorder. Participant retention and adherence were largely consistent with or higher than comparable studies. For example, only 6% of participants were lost at *t1*, which compares favorably to attrition rates of 21.6% (±16.9) reported in a recent meta-analysis of CBT for insomnia trials [54]. This may be due to the brevity of the intervention period, use of the Prolific recruitment platform (which is associated with high participant response rates), a focus on subclinical sleep disturbance, and reimbursing participants at each study time point. Similarly, 90.1% of participants accessed their allocated intervention and completed at least one session.

Post-intervention feedback ratings were largely positive, with 82.1% of participants reporting they were either “satisfied” or “very satisfied” with their allocated intervention, and 84.3% rating the quality of their intervention as either “good” or “excellent.” Ratings were similarly positive in the pragmatic waitlist group (who were given access to both interventions later). This is largely consistent with previous interventions evaluated on the Unmind MHapp [36]. Intervention engagement and feedback ratings were equivalent across the two audio tool categories, though participants in the pragmatic waitlist group mostly reported preferring one category over the other. This suggests that giving individuals choice over a wide variety of audio tools could facilitate intervention uptake and engagement.

Despite being encouraged to engage with the interventions each night over 4 weeks, participants completed an average of 12.7 separate sessions (with substantial variability across participants), and 44% completed the predefined threshold for per protocol engagement (12 sessions). As expected, engagement was lower in the pragmatic waitlist group, who were not given specific instructions and were free to engage as little or as often as desired. Unfortunately, we were unable to gather meaningful feedback in this study from participants in the waitlist group who chose not to use *Unmind*. Of those that did engage with the app, the primary barrier to engagement in both intervention arms and the waitlist group was forgetting to use the interventions, suggesting that future versions of the app (or a future definitive trial) should incorporate reminders. Participants also reported a range of other factors that impacted engagement, suggesting that tool usage is likely to naturally fluctuate over time in relation to individual circumstances.

Preliminary efficacy findings suggest that both interventions are associated with reductions in sleep disturbance and sleep-related impairment, with scores dropping from approximately 1 SD above population norms to normative levels, and between-group Hedge’s *g* effect sizes in the large range. This suggests that using the audio tools just a few times per week may be sufficient to experience significant improvements in sleep. Participants also reported sleeping an additional 35 minutes a night, with significant reductions in sleep onset latency post-intervention. This is broadly consistent with previous evidence that music can increase sleep quality and duration, as well as reduce sleep onset latency [24, 55], though this is one of the first RCTs to use a mobile app as the delivery medium, and to include narrated stories as an intervention arm.

In addition to improvements in sleep, the interventions also had a significant beneficial impact on self-reported symptoms of depression and anxiety, work- and activity-related impairment, and mental well-being, with effect sizes in the medium range. This is consistent with a recent meta-analysis reporting that interventions that improve sleep quality also result in medium-sized improvements across several mental health outcomes, such as depression, anxiety, stress, and composite measures of mental health [56], as well as with recent evidence that sleep is causally related to the experience of mental health difficulties [17]. Although the precise mechanisms underlying this relationship remain unclear, one potential explanation involves changes in aspects of emotion regulation. For example, previous research suggests that poor sleep can amplify the adverse effect of negative life events, dull the beneficial impact of positive events, and is associated with more frequent use of emotion regulation strategies that might be detrimental to good mental health [57, 58]. A future definitive trial should aim to explore these mechanisms with a reduced and more targeted set of secondary outcomes.

The audio tools evaluated in this study may have more widespread application and fewer barriers to adoption than more structured interventions, and may be helpful for individuals low in motivation (e.g. due to comorbid symptoms of depression or anxiety) since they do not require focused attention or learning new skills. Previous research suggests that the core active components of CBT for insomnia (which include sleep restriction therapy and stimulus control therapy) can be challenging, resulting in suboptimal adherence and reduced potential for treatment gains [59]. However, the present interventions are not intended as a replacement for CBT (or as a treatment for insomnia), and lower engagement levels in the pragmatic waitlist group suggest further research is needed to establish adherence patterns in both controlled and real-world settings. In addition, whilst these early findings suggest the study interventions may be efficacious in their own right, they could also have application as an adjunct to more structured treatment programs, and future research could explore this.

While this study did not explore the mechanisms via which the interventions improve sleep, it provides some preliminary support for the idea that listening to the tools might specifically shift attention away from inhibitory presleep cognitive processes (such as worrying about sleep, monitoring, or focusing on threat cues). This is because most participants reported using the tools as a means to fall asleep, and were more likely to engage with the tools on nights when they were feeling stressed or experiencing racing thoughts. However, this study did not directly investigate underlying mechanisms of action, and future research should aim to address this if we are to fully understand the potential impact and application of such tools.

To the best of our knowledge, this is the first study to demonstrate both feasibility and preliminary efficacy of app-based tools designed to aid sleep in a nonclinical sample. If these findings are replicated in a definitive trial, they could have widespread financial and public health implications for policymakers and employers. App-based audio tools such as those evaluated in this study are safe, affordable, widely accessible, and easy to use compared to structured interventions that require active learning or behavior change. Their wide availability means they may also be useful as an early intervention, preventing mild problems from becoming disordered, though further research is needed to test this hypothesis.

### Strengths and limitations

This study has several strengths. First, intervention adherence and engagement were objectively captured via the study app. Second, participant retention was high, reducing the potential impact of missing data on study findings. Third, all study outcomes and analyses were prospectively registered, precluding any selective reporting of outcomes or nonpublication of findings. Fourth, participants were screened for poor sleep using a well-validated measure of sleep disturbance. Fifth, participants were given access to a restricted version of the *Unmind* app, ensuring they only engaged with their allocated intervention. Sixth, we used an efficient multi-arm design to evaluate two separate but related interventions concurrently. Seventh, participants represented a wide age range of 18–64 years old.

One limitation of this study is that sleep quality and other mental health measures were captured immediately post-intervention with no follow-up period. Future research should aim to explore whether any outcome improvements are maintained over time, either with or without continued use of the interventions, as well as how frequently they need to be used to be beneficial. Such information could help to inform more meaningful per-protocol analyses in future RCTs, help to guide in-app engagement recommendations, and shed light on potential mechanisms of action. For example, if improvements in sleep following intervention use are short-lived, it might suggest that the audio tools predominantly act by counteracting presleep arousal or inhibitory cognitive processes, as opposed to changing underlying beliefs about sleep.

Similarly, we were unable to conduct exploratory dose–response analyses in this study, and could not test whether increased use of the study interventions predicts larger improvements in sleep. This should be explored in a fully powered trial if we are to understand the true impact of the interventions. We also used a passive control group as opposed to an active control group in which participants engage with activities matched for duration, attention, and interest. Passive controls can lead to inflated estimates of treatment effects, and do not account for nonspecific placebo effects. Although it is useful in practical terms to estimate this combined effect of the *Unmind* app, active controls are needed to estimate the specific impact and mechanisms underlying the study interventions. In addition, whilst we deliberately captured a broad set of mental health outcomes to estimate the scope of effects of this novel intervention type, a future definitive trial could use a more targeted set of outcomes, with less overlap between the constructs measured.

Another limitation is that participants were predominantly White and approximately 70% female (though females have a higher risk of developing sleep problems than males [9]), and thus future studies could aim to recruit more diverse and balanced study samples. Similarly, as with most web-based trials, participants were not blinded to group allocation. Finally, the participant pool was self-selected, and it remains unknown to what extent the present findings are generalizable to the wider working population.

## Conclusions

To the best of our knowledge, this is one of the first studies to evaluate the feasibility and preliminary efficacy of app-based audio tools designed to aid sleep in the general public. We show that such tools are feasible and may result in significant improvements in sleep quality, work productivity, and other mental health outcomes, when used by working adults without a sleep disorder. These findings were broadly replicated under both controlled and more pragmatic study conditions. Given their wide accessibility and ease of use, such interventions could have extensive financial and public health implications, and should be further investigated in a definitive RCT.

## Supplementary Material

zsad053_suppl_Supplementary_Material

zsad053_suppl_Supplementary_Data

## Data Availability

The data underlying this article will be shared on reasonable request to the corresponding author.

## Preprint Repositories

This manuscript has been submitted as a preprint on the PsyArXiv repository.
