# Supplementary material for: Feasibility and preliminary efficacy of app-based audio tools to improve sleep health in working adults experiencing poor sleep: a multi-arm randomized pilot trial
Source: Sleep. 2023 Mar 15;46(7):zsad053. doi: 10.1093/sleep/zsad053 (PMC10334735; doi:10.1093/sleep/zsad053)
Supplement: zsad053_suppl_Supplementary_Material [file zsad053_suppl_supplementary_material.docx]

Feasibility and preliminary efficacy of app-based audio tools to improve sleep

health in working adults experiencing poor sleep: a multi-arm

randomized pilot trial

Marcos Economides^1*^, Rhian Male^1^, Heather Bolton^1^, Kate Cavanagh^2^

1. Unmind Ltd, London, SE1 1LB, United Kingdom

2. University of Sussex, School of Psychology, East Sussex, BN1 9QH, United Kingdom

* corresponding author: marcos.economides@unmind.com

Table S1. Multi-select feedback ratings at *t1* (both intervention arms) and *t2* (WL control group).

|  | Study arm | | | |
| --- | --- | --- | --- | --- |
| Feedback question (n, %) | Overall | ST | NW | WL |
| How did you use the intervention sessions? |  |  |  |  |
| To listen to whilst falling asleep | 146 (53.7) | 68 (54.0) | 78 (53.4) | 57 (51.8) |
| To relax shortly or immediately before going to bed | 83 (30.5) | 40 (31.7) | 43 (29.5) | 34 (30.9) |
| To get back to sleep after waking up in the middle of the night | 40 (14.7) | 17 (13.5) | 23 (15.8) | 16 (14.5) |
| A different way | 3 (1.1) | 1 (0.8) | 2 (1.4) | 3 (2.7) |
| Reasons for not using the intervention more often? |  |  |  |  |
| Other | 67 (24.6) | 41 (28.7) | 26 (20.2) | 26 (26.0) |
| My sleep naturally improved | 43 (15.8) | 20 (14.0) | 23 (17.8) | 17 (17.0) |
| I had a change in circumstance that prevented me from using them more often (such as falling ill) | 38 (14.0) | 20 (14.0) | 18 (14.0) | 5 (5.0) |
| I didn’t feel like the intervention was helping | 33 (12.1) | 18 (12.6) | 15 (11.6) | 16 (16.0) |
| I didn’t have enough time | 32 (11.8) | 19 (13.3) | 13 (10.1) | 13 (13.0) |
| I experienced technical difficulties that prevented me from using it | 29 (10.7) | 12 (8.4) | 17 (13.2) | 10 (10.0) |
| I didn’t enjoy the intervention | 28 (10.3) | 12 (8.4) | 16 (12.4) | 11 (11.0) |
| I lost motivation to take part in the study | 2 (0.7) | 1 (0.7) | 1 (0.8) | 2 (2.0) |
| Reasons you were *more* likely to use the intervention on some nights compared to others? |  |  |  |  |
| Having a busy mind or racing thoughts | 95 (22.6) | 46 (21.8) | 49 (23.4) | 40 (23.4) |
| Not feeling tired enough or ready for sleep | 87 (20.7) | 50 (23.7) | 37 (17.7) | 27 (15.8) |
| Feeling more stressed or anxious than usual | 77 (18.3) | 32 (15.2) | 45 (21.5) | 41 (24.0) |
| Sleeping badly on previous nights | 68 (16.2) | 36 (17.1) | 32 (15.3) | 38 (22.2) |
| Wanting to drown out noise or external distractions | 55 (13.1) | 27 (12.8) | 28 (13.4) | 16 (9.4) |
| None of the above (no particular reasons) | 19 (4.5) | 8 (3.8) | 11 (5.3) | 3 (1.8) |
| Other reasons | 12 (2.9) | 7 (3.3) | 5 (2.4) | 3 (1.8) |
| Feeling less stressed or anxious than usual | 7 (1.7) | 5 (2.4) | 2 (1.0) | 3 (1.8) |
| Reasons you were *less* likely to use the intervention on some nights compared to others? |  |  |  |  |
| Forgetting to use them | 108 (25.5) | 56 (25.2) | 52 (25.9) | 49 (30.2) |
| Feeling naturally sleepy or ready for bed without any aids | 74 (17.5) | 38 (17.1) | 36 (17.9) | 27 (16.7) |
| Not having enough time on that night | 65 (15.4) | 32 (14.4) | 33 (16.4) | 0 (0.0) |
| A change in sleeping arrangement (such as staying overnight elsewhere, or a change in bed partner) | 44 (10.4) | 26 (11.7) | 18 (9.0) | 11 (6.8) |
| Practical reasons (such as not having Wi-Fi, not having headphones, or low phone battery) | 40 (9.5) | 19 (8.6) | 21 (10.4) | 23 (14.2) |
| Not feeling motivated to use them on that night | 29 (6.9) | 18 (8.1) | 11 (5.5) | 20 (12.3) |
| Feeling well-rested, or having slept well the night before | 24 (5.7) | 14 (6.3) | 10 (5.0) | 9 (5.6) |
| None of the above (no particular reasons) | 14 (3.3) | 6 (2.7) | 8 (4.0) | 6 (3.7) |
| Feeling less stressed or anxious than usual | 10 (2.4) | 5 (2.3) | 5 (2.5) | 12 (7.4) |
| Other reasons | 8 (1.9) | 3 (1.4) | 5 (2.5) | 2 (1.2) |
| Feeling more stressed or anxious than usual | 7 (1.7) | 5 (2.3) | 2(1.0) | 3 (1.9) |

Table S2. Raw, complete case scores at *t0* and *t1* for all intervention groups.

| Outcome | Estimates (mean, SE) | |
| --- | --- | --- |
|  | *t0* | *t1* |
| **SD-SF** |  |  |
| Control | 60.1 (0.57) | 59.0 (0.65) |
| Sleeptales | 59.4 (0.66) | 51.6 (0.93) |
| Nightwaves | 59.9 (0.64) | 50.9 (0.76) |
| **SRI-SF** |  |  |
| Control | 61.7 (0.71) | 59.7 (0.76) |
| Sleeptales | 61.5 (0.74) | 53.0 (1.08) |
| Nightwaves | 61.7 (0.72) | 52.3 (0.87) |
| **Sleep duration** |  |  |
| Control | 6.22 (0.12) | 6.27 (0.11) |
| Sleeptales | 6.35 (0.11) | 6.94 (0.11) |
| Nightwaves | 6.41 (0.11) | 6.99 (0.09) |
| **SWEMWBS** |  |  |
| Control | 19.7 (0.24) | 19.6 (0.31) |
| Sleeptales | 19.2 (0.31) | 21.3 (0.36) |
| Nightwaves | 20.1 (0.34) | 22.1 (0.37) |
| **PHQ-4** |  |  |
| Control | 4.55 (0.27) | 4.81 (0.34) |
| Sleeptales | 5.09 (0.34) | 3.70 (0.34) |
| Nightwaves | 4.82 (0.32) | 3.01 (0.27) |
| **Unmind Index** |  |  |
| Control | 93.1 (0.76) | 93.2 (0.97) |
| Sleeptales | 92.7 (0.99) | 100.9 (1.19) |
| Nightwaves | 94.6 (0.93) | 102.6 (1.03) |
| **WPAI** |  |  |
| *Absenteeism* |  |  |
| Control | 3.60 (0.75) | 3.87 (0.89) |
| Sleeptales | 3.54 (0.72) | 1.54 (0.58) |
| Nightwaves | 4.35 (1.52) | 1.39 (0.41) |
| *Presenteeism* |  |  |
| Control | 44.7 (2.20) | 40.6 (2.07) |
| Sleeptales | 44.7 (2.41) | 24.7 (2.27) |
| Nightwaves | 41.4 (2.36) | 29.5 (2.31) |
| *Total work impairment* |  |  |
| Control | 46.2 (2.28) | 42.1 (2.17) |
| Sleeptales | 46.4 (2.45) | 25.4 (2.37) |
| Nightwaves | 44.5 (2.46) | 30.2 (2.36) |
| *Activity impairment* |  |  |
| Control | 51.2 (2.21) | 46.9 (2.20) |
| Sleeptales | 52.6 (2.16) | 33.6 (2.53) |
| Nightwaves | 53.4 (2.34) | 32.3 (2.34) |

Table S3. Intervention feedback ratings from participants randomized to the WL control at follow-up (*t2*).

| Feedback question (n, %) | WL (n = 94) |
| --- | --- |
| Which intervention did you prefer? |  |
| Nightwaves | 30 (60.0) |
| Sleeptales | 17 (34.0) |
| No preference | 3 (6.0) |
| Was the Unmind app and intervention(s) easy to use? |  |
| No | 1 (1.4) |
| Useable after a lot of time and effort | 1 (1.4) |
| Useable after some time and effort | 4 (5.4) |
| Easy to learn | 31 (41.9) |
| Able to use app immediately | 37 (50.0) |
| Would you recommend the intervention(s) to people who might benefit from them? |  |
| Nobody | 1 (1.4) |
| Very few people | 12 (16.2) |
| Several people | 24 (32.4) |
| Many people | 17 (23.0) |
| Everyone | 20 (27.0) |
| Do you agree that the intervention(s) felt relevant to your personal situation and needs? |  |
| Strongly agree | 19 (25.7) |
| Agree | 41 (55.4) |
| Neither | 11 (14.9) |
| Disagree | 3 (4.1) |
| Strongly disagree | 0 (0.0) |
| Would you agree with the statement “I experienced lasting bad effects from use of the intervention(s)”? |  |
| Strongly agree | 0 (0.0) |
| Slightly agree | 1 (1.4) |
| Not sure | 3 (4.1) |
| Slightly disagree | 0 (0.0) |
| Strongly disagree | 70 (94.6) |
| How satisfied are you with the intervention(s)? |  |
| Very satisfied | 21 (28.4) |
| Satisfied | 35 (47.3) |
| Neither | 13 (17.6) |
| Dissatisfied | 5 (6.8) |
| Very dissatisfied | 0 (0.0) |
| How would you rate the quality of the intervention(s)? |  |
| Excellent | 23 (31.1) |
| Good | 35 (47.3) |
| Okay | 15 (20.3) |
| Poor | 1 (1.4) |

Table S4. Estimates marginal means (ITT sample) from linear mixed effects models with standardized effect sizes for the pragmatic wait-list group.

| Outcome | Estimates (mean, SE) | | | *P* value  (Within-group) | Hedges *g* [95% CI]  (Within-group) |
| --- | --- | --- | --- | --- | --- |
|  | *t1* | *t2* | *t1 – t2* |  |  |
| SD-SF | 59.0 (0.74) | 53.1 (0.76) | 5.91 (0.92) | < 0.001 | 0.78 [0.52 – 1.05] |
| SRI-SF | 59.9 (0.79) | 53.6 (0.80) | 6.29 (0.88) | < 0.001 | 0.79 [0.55 – 1.03] |
| Sleep duration | 6.25 (0.11) | 6.57 (0.12) | 0.32 (0.12) | 0.008 | 0.27 [0.08 – 0.47] |
| SWEMWBS | 19.6 (0.32) | 21.0 (0.32) | 1.38 (0.33) | < 0.001 | 0.43 [0.22 – 0.64] |
| PHQ-4 | 4.82 (0.32) | 3.57 (0.32) | 1.25 (0.31) | < 0.001 | 0.39 [0.20 – 0.59] |
| Unmind Index | 93.1 (1.02) | 99.2 (1.03) | 6.08 (1.02) | < 0.001 | 0.59 [0.38 – 0.80] |
| WPAI |  |  |  |  |  |
| *Absenteeism* | 3.88 (1.00) | 4.58 (1.02) | 0.69 (1.39) | 0.619 | 0.07 [-0.02 – 0.34] |
| *Presenteeism* | 41.0 (2.06) | 29.3 (2.06) | 11.6 (2.39) | < 0.001 | 0.56 [0.32 – 0.79] |
| *Total work impairment* | 42.4 (2.22) | 32.0 (2.25) | 10.4 (2.67) | < 0.001 | 0.46 [0.22 – 0.70] |
| *Activity impairment* | 47.2 (2.20) | 34.9 (2.23) | 12.2 (2.48) | < 0.001 | 0.55 [0.32 – 0.78] |
